# Delivery of Antigen to CD8^+^ Dendritic Cells by Fusing Antigen With Formyl Peptide Receptor-Like 1 Inhibitor Protein Induces Antitumor Immunity

**DOI:** 10.3389/fimmu.2019.01839

**Published:** 2019-08-02

**Authors:** Chen-Yi Chiang, Chiao-Chieh Wu, Yi-Jyun Chen, Shih-Jen Liu, Chih-Hsiang Leng, Hsin-Wei Chen

**Affiliations:** ^1^National Institute of Infectious Diseases and Vaccinology, National Health Research Institutes, Miaoli, Taiwan; ^2^Institute of Biotechnology, National Tsing Hua University, Hsinchu, Taiwan; ^3^Graduate Institute of Biomedical Sciences, China Medical University, Taichung, Taiwan; ^4^College of Medicine, Graduate Institute of Medicine, Kaohsiung Medical University, Kaohsiung, Taiwan

**Keywords:** antitumor immunity, dendritic cells, FLIPr, immunotherapy, targeted antigen

## Abstract

A major challenge for vaccine development is targeting antigens to dendritic cells (DCs) *in vivo*, enabling cross-presentation, and inducing the memory responses. Fcγ receptors (FcγRs) are expressed on many cell types including DCs. Therefore, targeting of antigen to DCs via FcγRs is an attractive strategy for vaccine development. This study employ formyl peptide receptor-like 1 inhibitory protein (FLIPr), an FcγR binding protein secreted by *Staphylococcus aureus*, to deliver antigen to DCs. Our results show that FLIPr is a competent vehicle in delivering antigen to CD8^+^ DCs for induction of potent immunities without extra adjuvant formulation. Fusion antigen with FLIPr enables effective antigen presentation on both MHC class II and class I to induce memory T cell responses. Altogether, using FLIPr as an antigen delivery vector has great potential to apply antigens for cancer immunotherapy as well as other infectious disease vaccines.

## Introduction

Antigen-presenting cells (APCs) are critical in the induction of immune responses. Professional APCs capture and process antigens in the peripheral tissue, express lymphocyte co-stimulatory molecules, migrate to lymphoid organs, secrete cytokines to initiate immune responses. Dendritic cells (DCs) are the most potent APCs. They play a critical role during the initiation of adaptive immune responses, which inducing differentiation of naïve T cells (CD4^+^ or CD8^+^ T cells) into effector cells (helper or cytotoxic T cells) and further regulation of humoral immune responses (1–3). These characters have made DCs as prime targets for immune-modulation strategies (4, 5).

DCs express various Fcγ receptors (FcγRs), which mediate internalization of antigen-antibody complexes (immune complexes, ICs) and regulate immune responses (6, 7). Several reports have shown that antibody-bound soluble antigens facilitate DCs to activate antigen-specific T cells more efficiently than free antigens (8–13). These results support that FcγRs play an important role in augmenting antigen presentation. Importantly, ICs not only enhance CD4^+^ T cell responses but also increase CD8^+^ T cell responses by entering cross-presentation pathway (9, 14–16). Activating and inhibitory FcγRs have been described in mice and human. Lehmann et al. (17) chose FcγR2b (an inhibitory receptor) and FcγR4 (an activating receptor) as model receptors. Both anti-FcγR2b-OVA and anti-FcγR4-OVA were able to induce T cell proliferation *in vivo* by targeting antigen to FcγR2b and FcγR4, respectively. In addition, they used NOTAM mice (the endogenous FcRγ-chain is replaced by a variant with a nonfunctional ITAM) to rule out the influence of possible ITAM-dependent signaling effects on the T cell responses induced by targeting of activating FcγRs. These results indicate that targeting antigens to FcγRs can induce T cell responses no matter activating or inhibitory FcγRs. Therefore, targeting of antigen to DCs via FcγRs potentially constitutes an effective strategy for induction of antigen-specific immune responses. However, using ICs is not practical because of increasing difficulties and costs of vaccine preparation.

Pathogens develop different ways to escape from the host immune responses. It has been demonstrated that *Staphylococcus aureus* evades FcγR-mediated immunity by secreting potent FcγR antagonists, such as formyl peptide receptor-like 1 inhibitory protein (FLIPr) or its homolog FLIPr-like (18). Both proteins can bind to FcγRs and inhibit IgG-mediated effector functions. Since FLIPr and FLIPr-like possess ability of binding to FcγRs, this character make FLIPr and FLIPr-like are potential vectors to deliver antigen to DCs via FcγRs and enhance immune responses.

Therefore, we hypothesized that FLIPr can guide antigen-FLIPr fusion protein to FcγRs increasing antigen uptake by APCs and facilitate antigen processing and presentation, then promote antigen-specific immune responses. To test this hypothesis, ovalbumin (OVA) was used as a model antigen. The merit of antigen-FLIPr fusion protein was validated by showing the accessibility to DCs, enhancement of antigen processing and presentation on both MHC class II and class I pathways, and induction of CD8^+^ T cell-mediated antitumor immunity without exogenous adjuvant formulation.

## Materials and Methods

### Reagents and Antibodies

Fluorochrome-conjugated antibodies specific for CD3e (145-2C11: FITC, PerCP-Cy5.5, BV510), CD4 (GK1.5: PerCP), CD8α (53-6.7: APC-Cy7, PerCP), CD11b (M1/70: PE-Cy7, BV421), CD11c (N418: APC-Cy7, BV421), CD19 (1D3: FITC, PE-Cy7), CD27 (LG.7F9: FITC), CD40 (3/23: APC), CD43 (1B11: PE-Cy7), CD127 (A7R34: PerCP-Cy5.5), Ly6C (HK1.4: PE-Cy7), Ly6G (1A8, FITC, BV421), MHCII (AF6-120, PerCP-C5.5), NK1.1 (PK136:FITC, PE), PDCA-1 (JF05-1C2.4.1: PE) were purchased from Biolegend, eBioSience, and BD. Other stains used were anti-mouse CD16/32 antibody, streptavidin-APC, streptavidin-BV510, streptavidin-alexa568, and streptavidin-alexa647. Live/Dead Fixable Green Dead Staining kit, for 488 nm excitation was purchased from Invitrogen and applied for flow cytometry discrimination of live and dead cells.

### Construction of Expression Vectors

Based on the amino acid sequence of OVA (accession number P0102) and FLIPr (accession number BAB57318), the DNA sequence encoding OVA-FLIPr were optimized for *Escherichia coli* codon usage and fully synthesized by Genomics Co. (New Taipei City, Taiwan). OVA-FLIPr DNA contained a linker sequence, encoding 4 glycines and 1 serine residue with three repeats (GGGGS)3, between OVA and FLIPr. The forward primer (5′- GGAATTCCATATGGGCAGCATTGGCGCGGCGAGCAT−3′, NdeI site is underlined) combined with reverse primer (5′- CACGAGCTCGAGATCCCAATAAATGCTATC 3′−3′, *Xho*I site is underlined) were used to amplify the synthetic DNA of OVA-FLIPr. The PCR product was then cloned into the *Nde*I and *Xho*I sites of the expression vector pET-22b(+) (Novagen, Madison, WI) to produce the plasmid pOVA-FLIPr. As a result, the C-terminus of rOVA-FLIPr contained a hexahistidine tag (His-tag). Construction of OVA expression vectors was described before (19).

### Production and Purificaton of rOVA and rOVA-FLIPr

To express protein, *E. coli* BL21 (DE3) (Invitrogen, Carlsbad, CA) was transformed with pOVA-FLIPr. The transformed cells were cultured at 37°C overnight. One 6-ml of the overnight culture was scaled up to 600 ml in a 2 L-shake flask and incubated at 37°C for 2.5 h before induction. Protein expression was induced (OD_600_ = 0.5) by adding 1 mM IPTG, followed by incubation at 37°C for 3 h. rOVA-FLIPr was purified by disrupting the harvested cells in a French press (Constant Systems, Daventry, UK) at 25 Kpsi in homogenization buffer [20 mM Tris (pH 8.0), 40 mM sucrose, 400 mM NaCl and 10% glycerol]. The cell lysate was clarified by centrifugation (32,000 rpm for 40 min). Most of the rOVA-FLIPr was present in inclusion bodies. rOVA-FLIPr was then solubilized with extraction buffer [20 mM Tris (pH 8.9), 40 mM sucrose, 400 mM NaCl, 10% glycerol, 20 mM Immidazole, and 6M guanidine hydrochloride]. The extracted fraction was loaded onto immobilized metal affinity chromatography (IMAC) columns (BIO-RAD, Hercules, CA, USA, 2.5 cm i.d. × 10.0 cm) containing 20 ml Ni-NTA resin (Qiagen, San Diego, CA, USA) to purify rOVA-FLIPr. The column washed with the extraction buffer and the same buffer containing 40 mM imidazole, and then washed with a 100-fold column volume of 10 mM Na_2_HPO_4_ and 0.4 M NaCl containing 0.1% Triton X-114 to remove the LPS. Next, the column was washed without 0.1% Triton X-114 to remove the residual detergent, and rOVA-FLIPr was eluted with 10 mM Na_2_HPO_4_ containing 500 mM imidazole. The eluted rOVA-FLIPr was dialyzed to 10 mM Na_2_HPO_4_ three times for at least 6 h each time. The endotoxin levels of the purified rOVA-FLIPr were determined by the Limulus amebocyte lysate (LAL) assay (Associates of Cape Cod, Inc., Cape Cod, MA), and the resulting endotoxin levels were <10 EU/mg. After dialysis against 50 mM Ammonia bicarbonate pH 8.0, the rOVA-FLIPr was lyophilized and stored at −20°C. The fractions from each step were analyzed by SDS-PAGE and immunoblotted with anti-His-tag antibodies. Preparation of rOVA was described before (19).

### FACS Analysis and Cell Sorting

Antibody staining followed by flow cytometry was performed to analyze cell surface marker expression. FACS buffer (PBS, 1%FBS, 1 mm EDTA, and 0.1% Sodium azide) was used in all FACS steps. Non-specific antibody binding via Fc receptors was blocked by cell staining with anti-mouse CD16/32 antibody at 4°C for 15 min. In the first staining step, cells were incubated with labeled antibodies at 4°C for 30 min. After washing, biotinylated antibodies were stained with fluorochrome-conjugated streptavidin at 4°C for 30 min. Flow cytometry was performed immediately on an Attenune NxT flow cytometer (Invitrogen CA, USA). Cell population were isolated by sorting following flow cytometry and fluorochrome-conjugated antibodies. After a final wash prior to sorting, cells were filtered through a 70-μm nylon strainer (BD) for removal of cell clumps. Cell sorts were performed on a FACS (BD FACS Influx Flow Cytometer). Sorting populations were collected in growing medium (10%FBS in RPMI). Data were analyzed with FlowJo_V10 software (Three Star: Ashland, OR, USA). The gating strategy of DC subpopulations are shown in Supplementary Figure 1.

### Mice

Female C57BL/6 mice were purchased from the National Laboratory Animal Center (Taipei, Taiwan). OT-1 (OVA_257−−264_ peptide-specific CD8^+^ TCR transgenic) and OT-2 (OVA_323−−339_ peptide-specific CD4^+^ TCR transgenic) mice were bred at the Laboratory Animal Center of the National Health Research Institutes (NHRI). Animals were housed in animal facilities of NHRI. All the animal studies were approved and were performed in compliance with the guidelines of the Animal Committee of the NHRI.

### Immunization

Groups of C57BL/6 mice (6–8 weeks of age) were immunized with rOVA or rOVA-FLIPr (30 μg/dose, unless indicated otherwise) via footpad injection. Mice immunized with PBS (without antigens) were used as controls. All animals were immunized once or 2 times at a 2-week interval with the same regimen.

### Capture Enzyme-Linked Immunosorbent Assays

Fcγ receptor-1 (ACROBiosystems DE, USA),−2b (Sino Biological Beijing, China),−3 (ACROBiosystems DE, USA), or−4 (Sino Biological Beijing, China) was coated on 96-well plates (0.5 μg/well). After blocking (5% skim milk in PBS), a 3-fold serial dilution (starting at 500 μM) of biotin-conjugated rOVA or rOVA-FLIPr was added to each well and incubated at room temperature for 2 h. The unbound rOVA or rOVA-FLIPr were washed then added HRP-conjugated streptavidin for the detection of binding protein. A substrate, 3, 3′, 5, 5′-tetramethylbenzidine (TMB), was added for color development. The absorbance was measured with an ELISA reader at 450 nm.

### *In vitro* Activation Assays

Splenocytes from OT-2 or OT-1 transgenic mice were labeled with CFSE (0.5 μM) at 37°C for 15 min. After washing, CFSE-labeled splenocytes were seeded into 24-well plates (5 × 10^6^ cells/well) and cultured in graded doses of rOVA-FLIPr or rOVA for 3 days. Cell cultured with OT-2 peptides, OT-1 peptides, or media alone were served as controls. Concentration of IL-2 and IFN-γ in the supernatants were measured by ELISA. Cells were harvested and stained with PerCP conjugated anti-CD4 or anti-CD8. The proliferation of CD4^+^ or CD8^+^ T cells were evaluated by flow cytometry for CFSE dilution.

### T Cell Activation by DC Subsets

CD4^+^ and CD8^+^ T cells from spleens of OT-2 or OT-1 transgenic mice were purified using CD4^+^ and CD8^+^ T cell isolation kits (Miltenyi Biotech.) following the manufacturer's instructions, respectively. To evaluate the T cell activation capacity of different DCs subsets, CD8^+^ DCs, CD8^−^ DCs, and pDCs from splenocytes of naïve C57BL/6 mice were isolated by FACS (BD FACS Influx Flow Cytometer) and used as APCs. Graded numbers of APCs in the presence of rOVA or rOVA-FLIPr (10 μg/ml) were cocultured with purified OT-2 CD4^+^ and OT-1 CD8^+^ T cells (5 × 10^5^ T cells/well) for 40 and 24 h, respectively. For some experiments, single suspension lymphocytes were prepared from lymph nodes which derived from mice injected with rOVA or rOVA-FLIPr. The CD11c^+^ and CD11c^−^ cells isolated by pan DC isolation kits or CD8^+^ DCs and CD8^−^ DCs isolated by FACS (BD FACS Influx Flow Cytometer) were used as APCs. Purified OT-2 CD4^+^ and OT-1 CD8^+^ T cells (5 × 10^5^ T cells/well) were cocultured with APCs without further addition of rOVA or rOVA-FLIPr for 40 and 24 h, respectively. Levels of IL-2 and IFN-γ in the supernatants were determined by ELISA.

### *In vitro* Binding Assays

Splenocytes were prepared from C57BL/6 mice. Cells were incubated in PBS containing biotin conjugated-rOVA or -rOVA-FLIPr on ice for 30 min. After washing, binding of rOVA or rOVA-FLIPr to CD8^+^ DCs, CD8^−^ DCs, and pDCs were detected by APC conjugated streptavidin and analyzed by flow cytometry. The gating strategy of DC subpopulations are shown in Supplementary Figure 1. The mean fluorescence intensity (MFI) for splenocytes incubated in PBS alone was defined as the basal level. The relative MFI was calculated by the formula: relative MFI = (MFI of cells incubated with rOVA or rVOA-FLIPr)/(MFI of cells incubated with PBS alone).

### Enzyme-Linked Immunospot (ELISPOT) Assays

The mice were sacrificed 3 or 17–19 weeks after the first immunization and splenocytes were prepared. The number of IFN-γ-producing cells in the spleen was evaluated using mouse IFN-γ ELISPOT kits (PB Pharmingen) according to the manufacturer's instruction. In brief, capturing antibodies were coated on 96-well plates with PVDF membranes (Millipore) and then incubated at 4°C for overnight. After washing with PBS, the plates were blocked with RPMI medium supplemented with fetal bovine serum (10%) for 1 h to prevent non-specific binding in later steps. The splenocytes (5 × 10^5^ cells/well) were seeded into the plates with OT-1 (OVA_257−−264_, SIINFEKL) and OT-2 (OVA_323−−339_, ISQAVHAAHAEINEAGR) peptides in triplicate wells. In parallel, concanavalin A (5 μg/mL), OT-1 control peptides (RAHYNIVTF, derived from human papillomavirus), OT-2 control peptides (GRLITVNPIVTEKDS, derived from dengue virus), and media (no stimulation) were included as controls. The splenocytyes were discarded from the plates by washing three times with 0.05% (w/v) Tween 20 in PBS after incubation at 37°C in a 5% CO_2_ humidified incubator for 2 days. The biotinylated detection antibody was added to wells (0.1 ml/well) then the plates were incubated at 37°C for 2 h. Repeating above washing steps and adding the avidin-horseradish peroxidase complex reagent, the plates were incubated at room temperature for 45 min. The plates were washed three times with 0.05% (w/v) Tween 20 in PBS and then three times with PBS alone. Staining solution (3-amine-9-ethylcarbazole, Sigma-Aldrich) was added to wells (0.1 ml/well) to develop the spots. After 1 h, the plates were placed under tap water to stop the reaction. The spots were determined by an ELISPOT reader (Cellular Technology Ltd., Shaker Heights, OH, USA).

### *In vivo* Killing Assays

To evaluate antigen-specific killing activity in the vaccinated mice *in vivo*, OT-1, and control peptide-pulsed syngeneic splenocytes were used as target cells in the killing assay. Splenocytes (2 × 10^7^ cells/ml) were incubated with OT-1 and control peptide at 37°C for 30 min, respectively. OT-1-pulsed splenocytes and control peptide-pulsed splenocytes were labeled with CFSE^high^ (5 μM) and CFSE^low^ (0.5 μM) at 37°C for 15 min. After washing, both cells were resuspended at 2 × 10^7^ cells/ml then mix at a 1:1 ratio in PBS prior to adoptive transfer into vaccinated mice via tail vein injection 1 week after the last immunization. The immunized mice were sacrificed 18 h after adoptive transfer and the killing of peptide-loaded splenocytes in spleen was analyzed by flow cytometry. The specific lysis was calculated using the following equation: % specific lysis = [1–(%CFSE^low^/%CFSE^high^) before injection/(%CFSE^low^/%CFSE^high^) after injection] × 100%.

### Tumor Model

EG7 cells (American Type Culture Collection, CRL-2113) were cultured in RPMI 1640 media supplemented with 10% (v/v) heat-inactivated fetal bovine serum, L-glutamine (2 mM), sodium pyruvate (1 mM), HEPES (10 mM), G418 (0.4 mg/ml), 2-mercaptoethanol (0.05 mM), and penicillin/streptomycin (50 units/mL) at 37°C under 5% CO_2_. Cells were harvested and washed with PBS. Mice were subcutaneously inoculated with 5 × 10^5^ or 5 × 10^4^ EG7 cells in 0.2 mL of PBS in the left flank as indication. Tumor growth was monitored by visual inspection and palpation. The tumor size was measured with a caliper, and the tumor volume was estimated by the formula V = width × length × (width + length)/2. The mice were sacrificed when the tumor volume reached 3,000 mm^3^. For some experiments, the mice were intraperitoneally treated anti-CD4, anti-CD8, or isotype control antibodies 1 day before tumor inoculation.

### Data Analysis

Values were expressed as mean ± SEM. The paired *t*-test was used to analyze the data between injected and non-injected sites of the same mouse. The Kruskal-Wallis test with Dunn's multiple comparison was used to compare differences for more than two groups. Statistical analysis was performed using GraphPad Prism software version 5.02 (GraphPad Software, San Diego, CA). Differences with *p* < 0.05 were considered to be statistically significant.

## Results

### Preparation and Functional Evaluation of rOVA-FLIPr

**Figure 1:**
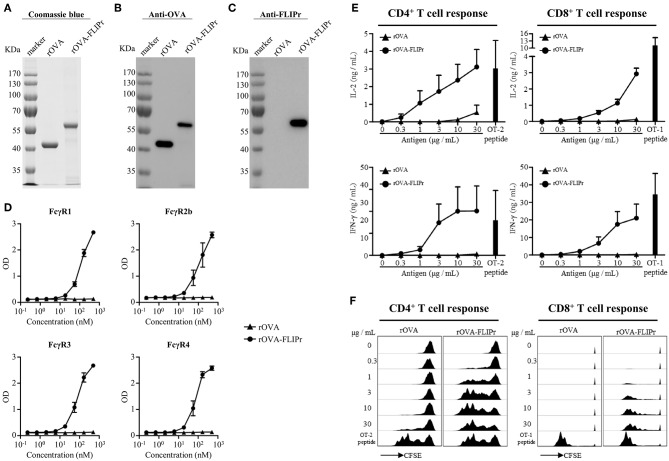
Production and characterization of rOVA and rOVA-FLIPr. Purified rOVA and rOVA-FLIPr were examined by 10% reducing SDS-PAGE followed by **(A)** Coomassie Blue staining and immunoblotting with **(B)** anti-OVA or **(C)** anti-FLIPr antibodies. **(D)** rOVA-FLIPr but not rOVA can bind to various Fcγ receptors. Fcγ receptor-1, −2b, −3, or −4 was coated on 96-well plates (0.5 μg/well). After blocking, graded concentration of biotin-conjugated rOVA or rOVA-FLIPr was added to each well and incubated at room temperature for 2 h. The unbound rOVA or rOVA-FLIPr were washed then added HRP-conjugated streptavidin for the detection of binding protein. A substrate, TMB, was added for color development. The absorbance was measured with an ELISA reader at 450 nm. The results are pooled from two independent experiments with a total 4 wells. rOVA-FLIPr elicits stronger T cell activation than rOVA alone in a dose-dependent manner. CFSE-labeled splenocytes from OT-2 or OT-1 transgenic mice were cultured in graded doses of rOVA-FLIPr or rOVA for 3 days. The supernatants were harvested. **(E)** IL-2 or IFN-γ production were measured by ELISA. Data represent mean ± SEM from 4 independent experiments. **(F)** The proliferation of CD4^+^ or CD8^+^ T cells were evaluated by flow cytometry for CFSE dilution. Data are representative of 3 experiments.

To examine the potential of FLIPr delivered antigen for T cell activation, we produced recombinant OVA (rOVA) and recombinant OVA-FLIPr fusion protein (rOVA-FLIPr) from an *Escherichia coli*-based system. Purified rOVA and rOVA-FLIPr were examined by 10% reducing SDS-PAGE followed by Coomassie Blue staining (Figure 1A) and further confirmed by immunoblotting with anti-OVA or anti-FLIPr antibodies. The anti-OVA antibodies recognized both rOVA and rOVA-FLIPr (Figure 1B). However, the anti-FLIPr antibodies only recognized rOVA-FLIPr but not rOVA (Figure 1C). These results suggest that the purified recombinant proteins are rOVA and rOVA-FLIPr, respectively. To analyze the functional activity of rOVA-FLIPr, a capture ELISA was performed to confirm that rOVA-FLIPr directly interact with different FcγR isoforms. As shown in Figure 1D, rOVA-FLIPr was captured by FcγR1, FcγR2b, FcγR3, and FcγR4. In contrast, there was no interaction between rOVA and different FcγR isoforms we tested. These results indicate that rOVA-FLIPr possesses the ability of binding to different FcγR isoforms.

We next evaluated the capacity of rOVA-FLIPr to deliver the fused antigens into MHC class II and MHC class I antigen presentation pathways eventually leading to the activation of CD4^+^ or CD8^+^ T cells. Therefore, CFSE-labeled splenocytes from OT-2 or OT-1 TCR transgenic mice were cultured by stimulation of different amounts of rOVA or rOVA-FLIPr for 3 days. OT-2 and OT-1 peptides were served as positive controls for CD4^+^ and CD8^+^ T cell activation, respectively. It is evident for both CD4^+^ and CD8^+^ T cells that rOVA-FLIPr induce higher levels of IL-2 and INF-γ production (Figure 1E) as well as T cell proliferation (Figure 1F) than rOVA alone in a dose-dependent manner.

### Induction of Memory T Cell Responses and Recall Activity by Immunization of Mice With rOVA-FLIPr

**Figure 2:**
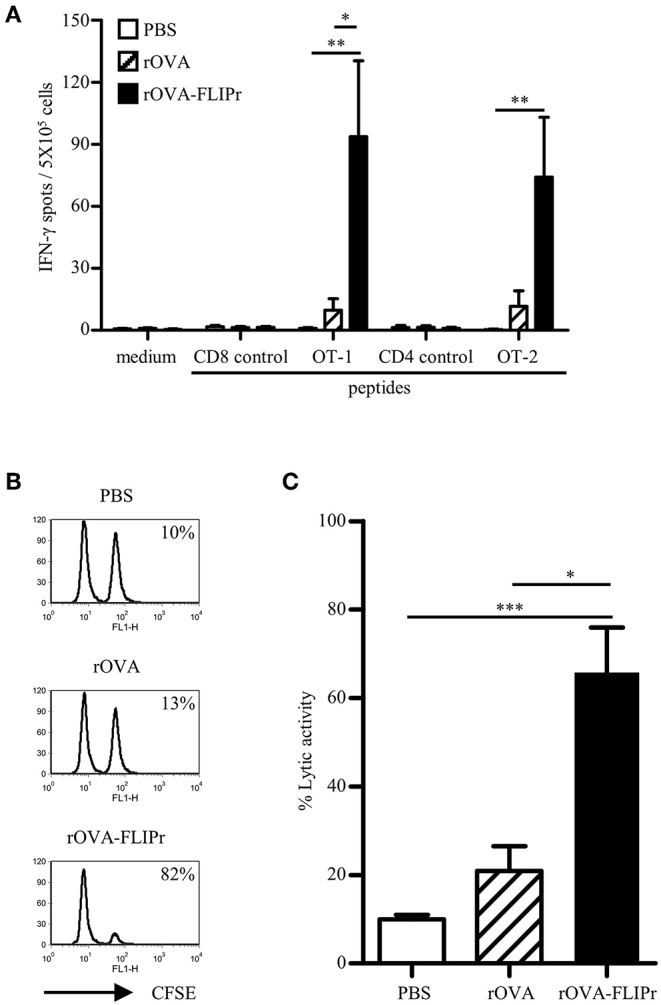
Immunization of mice with rOVA-FLIPr enhances CD4^+^ and CD8^+^ T cell. Groups of C57BL/6 mice were immunized twice with 30 μg of rOVA or rOVA-FLIPr at a 2-week interval. Mice immunized with PBS were used as controls. **(A)** One week after the last immunization, splenocytes were incubated with OT-1, OT-2, or control peptides for 48 h in an anti-INF-γ-coated 96-well ELISPOT plate. The IFN-γ producing spots were determined using an ELISPOT reader. Results are expressed as the mean ± standard errors of the mean (*n* = 6, pooled from two independent experiments). **(B)** An equal mixture of OT-1 peptide-pulsed splenocytes (CFSE^high^) and control peptide-pulsed splenocytes (CFSE^low^) were injected into the immunized mice via intravenous routes. The immunized mice were sacrificed 18 h later and the killing of peptide-loaded splenocytes in spleen was analyzed by flow cytometry. Representative profiles of mice from each group are shown. **(C)** *in vivo* cytotoxic T lymphocyte killing was calculated by the formula: % specific lysis = [1–(%CFSE^low^/%CFSE^high^) before injection/(%CFSE^low^/%CFSE^high^) after injection] × 100%. Bars represent the mean percentage of specific lysis ± SEM in each group (*n* = 8, pooled from two independent experiments). The statistical significance was determined using the Kruskal-Wallis test with Dunn's multiple comparison test. ^*^p < 0.05, ^**^p < 0.01, and ^***^p < 0.001.

To ascertain whether the *in vitro* activity of rOVA-FLIPr associate with the immune responses *in vivo*, we immunized mice with rOVA or rOVA-FLIPr and evaluated the immune responses after immunization. The animals immunized with PBS alone served as negative controls. One week after the last immunization, the splenocytes were examined for the frequencies of IFN-γ-secreting cells. Mice immunized with rOVA-FLIPr induced high frequencies of IFN-γ-secreting cells after stimulation with OT-2 (a CD4-epitope) or OT-1 (a CD8-epitope) peptides. In contrast, only low frequencies of IFN-γ-secreting cells were obtained in the splenocytes of rOVA immunized mice. Background levels of IFN-γ-secreting cells were detected from all of the splenocytes without stimulation (medium alone) or stimulated with control peptides (Figure 2A). Furthermore, an *in vivo* killing assay was conducted to determine the killing activity induced by rOVA-FLIPr or rOVA. Mice immunized with rOVA-FLIPr induced superior killing activities to mice immunized with rOVA (Figures 2B,C). In conclusion, fusion OVA with FLIPr can enhance OVA-specific CD4^+^ and CD8^+^ T cell responses.

**Figure 3:**
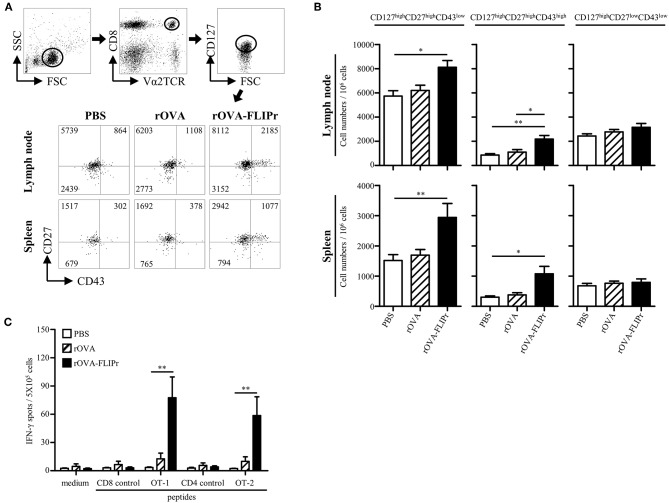
Induction of memory T cell responses with recall activity by immunization of mice with rOVA-FLIPr. **(A)** Groups of C57BL/6 mice were immunized once with rOVA or rOVA-FLIPr (5 μg/hind foot pad). Mice immunized with PBS were used as controls. OT-1 CD8^+^ T cells (5 × 10^5^) were adoptively transferred via the tail vein 1 day before immunization. Lymphocytes from lymph nodes (popliteal and inguinal) and spleens were isolated at 28 days after immunization. OT-1 cells (CD8^+^V2αTCR) with CD127^high^ phenotype (a memory T cell marker) were gated for analyzing CD27 and CD43 expression. Representative profiles of mice from each group are shown. **(B)** Results are expressed as the mean ± standard errors of the mean (*n* = 8–9, pooled from three independent experiments). **(C)** Groups of C57BL/6 mice were immunized twice with 30 μg of rOVA or rOVA-FLIPr at a 2-week interval. Mice immunized with PBS were used as controls. Spleen were removed at 17–19 weeks after the first immunization. Splencoytes were prepared and incubated with OT-1, OT-2, or control peptides for 48 h in an anti-INF-γ-coated 96-well ELISPOT plate. The IFN-γ producing spots were determined using an ELISPOT reader. Results are expressed as the mean ± SEM of the mean (*n* = 6, pooled from two independent experiments). The statistical significance was determined using the Kruskal-Wallis test with Dunn's multiple comparison test. ^*^p < 0.05 and ^**^p < 0.01.

Induction of memory immune responses is crucial for vaccine development as well as cancer immunotherapy. To verify the memory CD8^+^ T cell profiles after immunization, we used a memory T cell marker (CD127) (20, 21) and activation markers (CD27 and CD43) (22). OT-1 cells were adoptively transferred 1 day before immunization, then, the lymphocytes from lymph nodes (popliteal and inguinal) and spleens were harvested on day 28 after immunization. OT-1 cells with CD127^high^ phenotypes were gated for analyzing CD27 and CD43 expression by flow cytometry. Three main populations were observed: CD127^high^ CD27^high^ CD43^low^, CD127^high^ CD27^high^ CD43^high^, and CD127^high^ CD27^low^ CD43^low^ subsets (Figure 3A) which represent recall capacity of memory T cells in terms of stimulation from high to low (22). The CD127^high^ CD27^high^ CD43^low^ and CD127^high^ CD27^high^ CD43^high^ subsets were significantly increased in the rOVA-FLIPr immunized mice but not in the rOVA or PBS groups in both the lymph nodes and spleens (Figure 3B). In contrast, there was no significant difference in the CD127^high^ CD27^low^ CD43^low^ subset among these groups (Figure 3B).

To examine recall activity of antigen-specific memory T cells *in vivo*, we immunized mice with PBS, rOVA, or rOVA-FLIPr and evaluated the frequencies of IFN-γ-secreting cells on week 17–19 after immunization (Figure 3C). Consistently, high frequencies of IFN-γ-secreting cells were still detected after stimulation with OT-2 (a CD4-epitope) or OT-1 (a CD8-epitope) peptides in mice immunized with rOVA-FLIPr, but not mice immunized with rOVA or PBS. Background levels of IFN-γ-secreting cells were detected from all of the splenocytes without stimulation (medium alone) or stimulated with control peptides. These results suggest that rOVA-FLIPr immunization is able to elicit long-lived memory T cell responses.

### Inhibition of Tumor Growth by Treatment With rOVA-FLIPr

**Figure 4:**
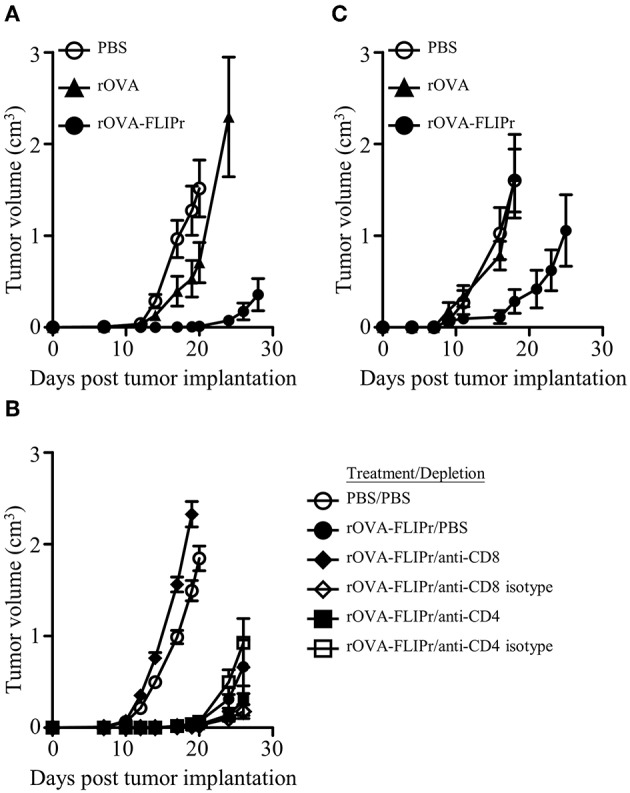
Immunization of mice with rOVA-FLIPr induces CD8^+^ T cell-mediated antitumor immunity. Groups of C57BL/6 mice (*n* = 6/group) were immunized twice with 30 μg of rOVA or rOVA-FLIPr at a 2-week interval. Mice immunized with PBS were used as controls. One week after the last immunization, mice were implanted with EG7 cells (5 × 10^5^). **(A)** Tumor growth was monitored. The results are one of two representative experiments. **(B)** One day before tumor implantation, mice were intraperitoneally injected with anti-CD4 antibodies, anti-CD8 antibodies, or their isotype control antibodies. Mice immunized with PBS and depleted with PBS were used as controls. Tumor growth was monitored after depletion. **(C)** Mice were inoculated EG7 cells (5 × 10^4^) on day 0 and immunized with 30 μg of rOVA or rOVA-FLIPr on day 3 and day 10. Mice immunized with PBS were used as controls. Tumor growth was monitored. The results are one of two representative experiments.

Given the induction of superior immune responses elicited by vaccination with rOVA-FLIPr, we evaluated the vaccinated mice for an *in vivo* antitumor effect against challenge by EG7 cells, derived from EL4 (a mouse lymphoma cell line) transfected OVA gene and produce OVA constitutively. One week after the last vaccination, animals were challenged with EG7 cells. Tumor growth was inhibited in mice immunized with rOVA-FLIPr (Figure 4A). These results indicate that in a preventative vaccination setting, rOVA-FLIPr can induce antitumor responses that reduce tumor growth *in vivo*. The antitumor effect in rOVA-FLIPr-immunized mice was abolished in anti-CD8 antibody-depleted mice but not anti-CD4- or isotype antibody-depleted mice (Figure 4B). These results suggest that the CD8 population is mainly responsible for mediating *in vivo* antitumor responses in rOVA-FLIPr-immunized mice. We next evaluate the therapeutic potential of antigen fusion with FLIPr. Tumor-bearing mice were treated with rOVA, rOVA-FLIPr, and PBS on days 3 and 10 after inoculation of EG7 cells. Again, tumor growth was inhibited in mice immunized with rOVA-FLIPr. There was no benefit of mice treated with rOVA in comparison to PBS (Figure 4C).

### Targeting of rOVA-FLIPr to Dendritic Cells and Increasing the Efficiency of Antigen Presentation on Both MHC Class II and Class I

**Figure 5:**
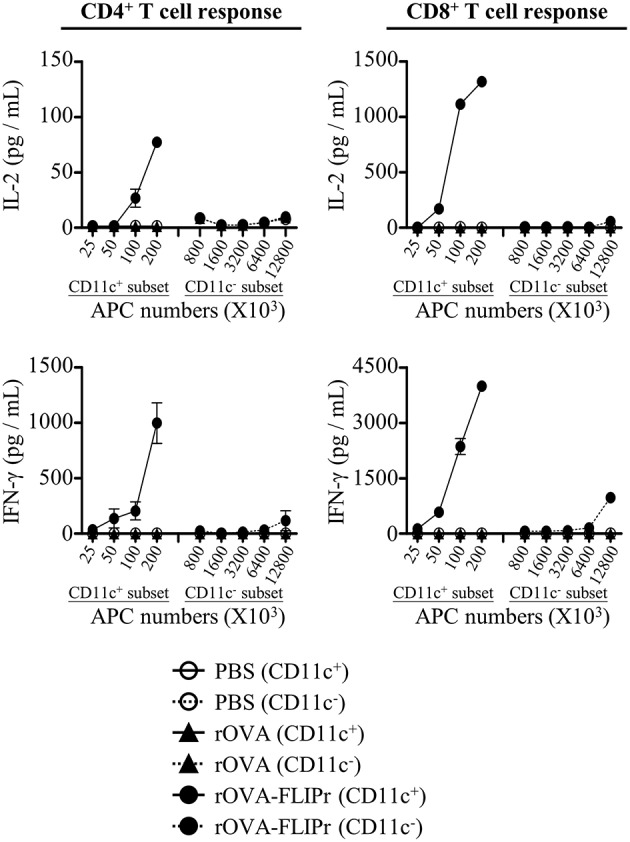
CD11c^+^ dendritic cells efficiently mediate rOVA-FLIPr induction of T cell activation. Groups of C57BL/6 mice were injected with rOVA, rOVA-FLIPr in the hind foot pads (50 μg/foot pad). Mice injected with PBS were used as controls. Lymph nodes were harvested 48 h after injection. Cells were divided into CD11c^+^ and CD11c^−^ subsets by anti-mouse CD11c magnetic beads. Graded numbers of each subset were cocultured with OT-2 and OT-1 cells for 24 and 40 h, respectively. Levels of IL-2 and IFN-γ in the supernatants were determined by ELISA. Representative results (mean ± SEM) of 2 separate experiments performed in duplicate wells.

DCs are the most potent professional APCs. To examine whether antigen was delivered to DCs *in vivo*, we isolated CD11c^+^ and CD11c^−^ cells from draining lymph nodes 72 h after injection of mice with 50 μg rOVA-FLIPr, rOVA, or PBS. Different numbers of CD11c^+^ or CD11c^−^ cells were cocultured with CD4^+^ MCH class II-restricted OT-2 and CD8^+^ MCH class I-restricted OT-1 T cells without further addition of antigen for 40 and 24 h, respectively. Production of IL-2 and IFN-γ were determined by ELISA. The CD11c^+^-enriched subset obtained from draining lymph nodes of rOVA-FLIPr injected mice could stimulate OT-2 and OT-1 T cells to produce IL-2 and IFN-γ (Figure 5). Cytokine levels were increased as increasing the number of CD11c^+^-enriched cells. The CD11c^−^ subset was inefficient in activation of OT-2 and OT-1 T cells to produce IL-2 and IFN-γ. At least a 128-fold higher number of APCs was necessary to induce a comparable IL-2 or IFN-γ production. In contrast, neither CD11c^+^ nor CD11c^−^ cells obtained from draining lymph nodes of rOVA or PBS injected mice were able to activate OT-2 and OT-1 T cells. These results suggest that rOVA-FLIPr is efficient delivery to DCs for processing and presentation *in vivo*.

To demonstrate that targeting rOVA-FLIPr to DCs for T cell activation is via FcγRs, we used rabbit F(ab)'_2_ antibodies against FcγR1, 2b, 3, or 4 to block FcγR binding. CD11c^+^ cells were treated with or without anti-FcγR antibodies before adding rOVA-FLIPr, then cocultured with OT-2 and OT-1 cells for 40 and 24 h, respectively. As shown in Supplementary Figure 2, IL-2 and IFNγ production were reduced in the presence of anti-FcγR antibodies. These results suggest that targeting rOVA-FLIPr to DCs for T cell activation is via FcγRs.

**Figure 6:**
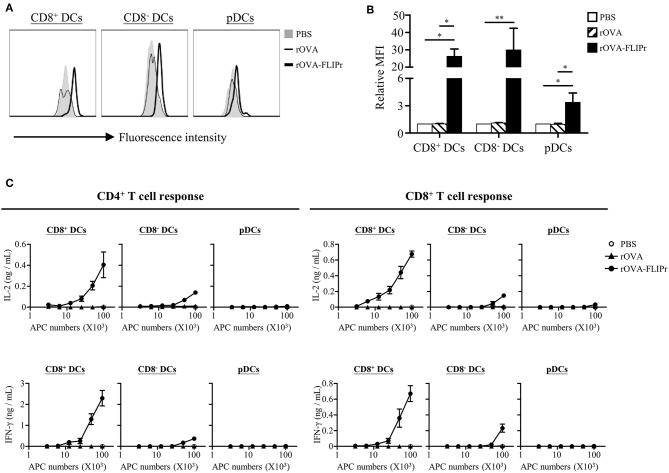
Targeting of rOVA-FLIPr to dendritic cell subpopulation *in vitro*. **(A)** Binding of rOVA or rOVA-FLIPr to the indicated DC populations. Splenocytes were incubated in PBS containing biotin conjugated-rOVA or -rOVA-FLIPr on ice for 30 min. After washing, binding of rOVA or rOVA-FLIPr were detected by APC conjugated streptavidin and analyzed by flow cytometry. **(B)** The mean fluorescence intensity (MFI) for cells incubated in PBS alone was defined as the basal level. The relative MFI was calculated by the formula: relative MFI = (MFI of cells incubated with rOVA or rVOA-FLIPr)/(MFI of cells incubated with PBS alone)]. The mean ± standard error from four independent experiments are shown. The statistical significance was determined using the Kruskal-Wallis test with Dunn's multiple comparison test. ^*^p < 0.05 and ^**^p < 0.01. **(C)** Graded numbers of sorted CD8^+^ DCs, CD8^−^ DCs, and pDCs were cocultured with OT-2 and OT-1 cells for 40 and 24 h, respectively. Levels of IL-2 and IFN-γ in the supernatants were determined by ELISA. Representative results (mean ± SEM) of 2 separate experiments performed in duplicate wells.

CD8^+^ DCs, CD8^−^ DCs, and plasmacytoid DCs (pDCs) are the three main lymphoid DC populations (23). To verify the capacity of antigen delivered by rOVA and rOVA-FLIPr to different DC subsets, we conjugated rOVA and rOVA-FLIPr with biotin. Splenocytes were incubated in PBS containing biotin conjugated-rOVA or -rOVA-FLIPr on ice for 30 min. Binding of rOVA or rOVA-FLIPr were detected by APC conjugated streptavidin and analyzed by flow cytometry. We found that rOVA-FLIPr, but not rOVA, bound to CD8^+^ DCs, CD8^−^ DCs, and pDCs (Figures 6A,B). In addition, rOVA-FLIPr was more efficient bound to CD8^+^ DCs and CD8^−^ DCs than pDCs.

Furthermore, we sorted CD8^+^ DCs, CD8^−^ DCs, and pDCs from spleen then treated them with rOVA or rOVA-FLIPr *in vitro*. Different numbers of DCs were cocultured with OT-2 and OT-1 T cells for 40 and 24 h, respectively. IL-2 and IFN-γ production levels were determined by ELISA. We found that OVA was presented by CD8^+^ DCs and CD8^−^ DCs, but not pDCs, on both MHC class II and class I products when DCs treated with rOVA-FLIPr (Figure 6C). In addition, we noticed that CD8^+^ DCs were superior to CD8^−^ DCs in activation of both CD4^+^ and CD8^+^ T cells when comparison to cytokine levels activated by the same DC numbers. In contrast, rOVA treated CD8^+^ DCs, CD8^−^ DCs, and pDC were unable to efficiently activate CD4^+^ and CD8^+^ T cells. These results suggest that rOVA-FLIPr increase the efficiency of OVA presentation on both MHC class II and class I relative to rOVA.

### Targeting of rOVA-FLIPr to CD8^+^ Dendritic Cells to Mediate CD4^+^ and CD8^+^ T Cell Activation

**Figure 7:**
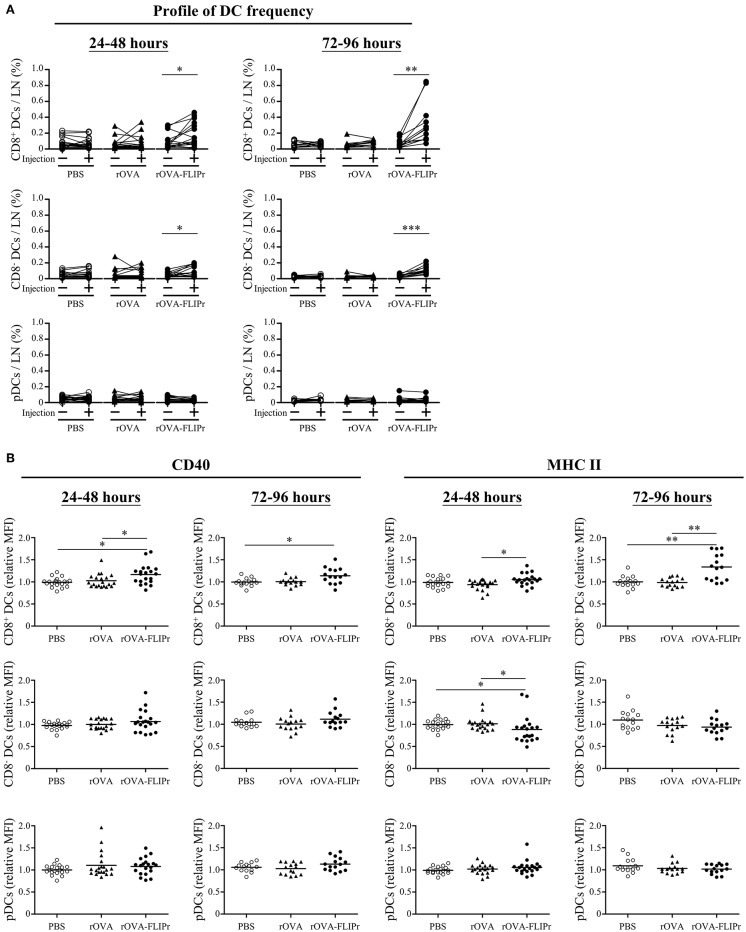
Injection of rOVA-FLIPr increases the frequency and maturation of CD8^+^ dendritic cells in lymph nodes. Groups of C57BL/6 mice were injected with rOVA, rOVA-FLIPr (100 μg) in the left hind foot pad. Mice injected with PBS were used as controls. Right (injection:–) and left (injection: +) inguinal lymph nodes were harvested 24–48 or 72–96 h after injection. **(A)** The frequencies of CD8^+^ DCs, CD8^−^ DCs, and pDC were analyzed by flow cytometry. **(B)** The expression of CD40 and MHC II on the surface of CD8^+^ DCs, CD8^−^ DCs, and pDC were analyzed by flow cytometry. The relative mean fluorescence intensity (MFI) of each mouse was calculated by the formula: relative MFI = (MFI of cells obtained from left (injection: +) inguinal lymph node)/(MFI of cells obtained from right (injection: –) inguinal lymph node). The results were pooled from 2 to 3 independent experiments for each time points. The statistical significance was determined by a paired *t*-test. ^*^p < 0.05, ^**^p < 0.01, and ^***^p < 0.0001.

Lymph nodes are the critical sites where DCs cross-talk with T cells to coordinate adaptive immune responses. To investigate the effect of rOVA-FLIPr on DCs at lymph nodes, groups of mice were injected with PBS, rOVA, or rOVA-FLIPr in their left hind foot pads. The frequencies of CD8^+^ DCs, CD8^−^ DCs, and pDC in both the left (the injected site) and right (the non-injected site) inguinal lymph nodes were examined by flow cytometry at 24–48 or 72–96 h after injection. Injection of rOVA-FLIPr increased the frequencies of CD8^+^ DCs and CD8^−^ DCs, but not pDCs, in the left inguinal lymph nodes in comparison to the right inguinal lymph node in the same mouse after injection (Figure 7A). Next, we analyzed expression of CD40 and MHC class II markers on different DC subsets. CD40 or MCH class II mean fluorescent intensities (MFI) of CD8^+^ DCs, CD8^−^ DCs, and pDC in the left inguinal lymph nodes were normalized by the right inguinal lymph node in the same mouse. Both CD40 and MHC class II expression levels of CD8^+^ DCs were elevated in the lymph node of rOVA-FLIPr injected sites (Figure 7B, upper panel). The CD40 relative MFI of CD8^−^ DCs were not significant different among PBS, rOVA, and rOVA-FLIPr injection groups, but MCH class II relative MFI of CD8^−^ DCs in the lymph node of rOVA-FLIPr injected sites were decreased at 24–48 after injection (Figure 7B, middle panel). There were no significant differences in CD40 and MCH class II relative MFI of pDCs among PBS, rOVA, and rOVA-FLIPr injection groups (Figure 7B, lower panel). These results indicate that injection of rOVA-FLIPr elevate CD40 and MCH class II expression of CD8^+^ DCs but not CD8^−^ DCs, and pDCs.

**Figure 8:**
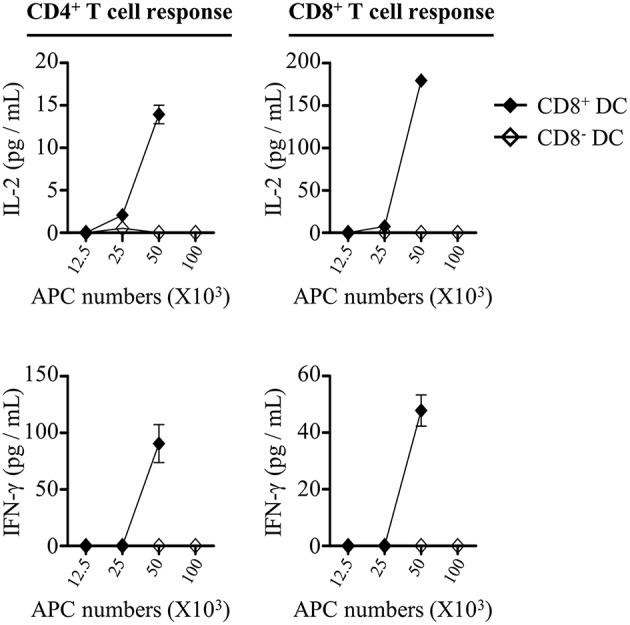
CD8^+^ dendritic cells can mediate rOVA-FLIPr induction of T cell activation *in vivo*. C57BL/6 mice were injected with rOVA-FLIPr in the hind foot pads (50 μg/foot pad). Lymph nodes were harvested 72 h after injection. Graded numbers of sorted CD8^+^ DCs and CD8^−^ DCs were cocultured with OT-2 and OT-1 cells for 24 and 40 h, respectively. Levels of IL-2 and IFN-γ in the supernatants were determined by ELISA. Representative results (mean ± SEM) of 2 separate experiments performed in duplicate wells.

Although the frequencies of CD8^+^ and CD8^−^ DCs in draining lymph nodes were elevated after injection of rOVA-FLIPr, only CD8^+^ DCs upregulated CD40 and MHC II expressions. We wanted to determine whether the differential effects on CD8^+^ and CD8^−^ DCs were associated with their capability to initiate CD4^+^ and CD8^+^ T cell responses *in vivo*. To address this question, we sorted CD8^+^ and CD8^−^ DCs from draining lymph nodes 72 h after injection of mice with 50 μg rOVA-FLIPr. Different numbers of CD8^+^ or CD8^−^ DCs were cocultured with OT-2 and OT-1 T cells without further addition of antigen for 40 and 24 h, respectively. Within the range of APC numbers we tested, only CD8^+^ DCs were capable of triggering OT-2 and OT-1 cells activation to produce IL-2 and IFN-γ (Figure 8). Overall, these data support that rOVA-FLIPr can target to CD8^+^ DCs then induce a CD4^+^ and CD8^+^ T cell response *in vivo*.

## Discussion

Modern vaccine designs have shifted from live attenuated or inactivated whole-pathogen vaccines to more pure and defined subunit vaccines. However, recombinant protein-based antigens have poor immunogenicity. To overcome this issue, subunit vaccines have to be administered in a suitable delivery system or adjuvant formulation. Targeting antigens to DC receptors *in vivo* provides an efficient approach to induce robust immune responses (4, 5). FcγRs are attractive receptors expressed on the surface of DCs which can be used as targets for antigen delivery. FLIPr, secreted by *Staphylococcus aureus*, can bind to FcγRs (18). Employing this property of FLIPr, we demonstrate that FLIPr is a potential vehicle for targeting antigen to DCs via FcγRs. In this study, we show that rOVA-FLIPr but not rOVA binds to FcγRs (Figure 1D). DCs (CD11c^+^-enriched subset) in the lymph nodes of injected sites obtained from mice immunized with rOVA-FLIPr can stimulate OT-2 and OT-1 T cell activation to produce IL-2 and IFN-γ. Furthermore, neither DCs obtained from mice immunized with rOVA nor PBS are able to activate OT-2 and OT-1 (Figure 5). These results support that rOVA-FLIPr can be targeted to DCs then trigger T cell activation *in vivo*.

DCs are a heterogeneous population, which consist of various subsets. CD8^+^ DCs, CD8^−^ DCs (belongs to the conventional DC population), and pDCs are the three main DC subsets in lymphoid tissues (23). They share many common antigen presentation features but have distinct functional specializations (24). OVA fusion with FLIPr is efficiently targeted to the three DC subsets (Figures 6A,B). It has been shown that CD8^+^ DCs and CD8^−^ DCs express all four FcγRs, pDCs solely express FcγRIIB (17). The amounts of targeted rOVA-FLIPr associate with the expression profiles of FcγRs on the DC surface (Figures 6A,B). When targeting antigens to DCs, the consequence of the immune response induced by DCs is dependent on several factors of which some are related to the DC subset that is target, whereas others are associated to the choice of target receptor (25). The DC subsets possess different capacities for antigen processing and presentation (26). Several studies (10, 27–30) reveal that CD8^+^ DCs and CD8^−^ DCs differ in their ability at cross-presentation of antigen to CD8^+^ T cells and activation of CD4^+^ T cells. In this study, we show that CD8^+^ DCs surpass CD8^−^ DCs and pDCs in activation of both CD4^+^ and CD8^+^ T cells when delivery of antigen by fusing antigen with FLIPr (Figures 6C, 8). The discrepancy capacity of T cell activation among DC subsets may due the intrinsic property of DC subsets or the effects of rOVA-FLIPr. Immunization of rOVA-FLIPr elevates the frequency of CD8^+^ DCs and expression levels of CD40 and MCH II on the CD8^+^ DCs in the draining lymph nodes of injected sites (Figure 7). These results indicate that CD8^+^ DCs are skewed to mature after rOVA-FLIPr injection. Even though the frequency of CD8^−^ DCs is increased in the draining lymph nodes of injected sites after immunization of rOVA-FLIPr, the MHC II expression decreases on the CD8^−^ DCs and CD40 expression still maintains at the similar levels (Figure 7). These results suggest that CD8^−^ DCs are not toward a maturation phenotype and do not favor T cell activation after rOVA-FLIPr injection. In contrast, immunization of rOVA-FLIPr does not change the frequency and activation status of pDCs *in vivo* (Figure 7). Besides, pDCs have little capacity of T cell activation when pDCs stimulate with rOVA-FLIPr *in vitro* (Figure 6C). These results suggest that pDCs might not be critical for rOVA-FLIPr-mediated induction of T cell responses. Our findings may reflect differences in the ability of CD8^+^ DC, CD8^−^ DC, and pDC subsets to stimulate immune responses *in vivo*.

Injection of antigen-targeting antibodies (anti-DEC205, anti-DCIR2, or anti- FcγRs) does not change activation status of DCs *in vivo* (17, 31). When delivery of antigen to DCs in the steady state induces transient but not long-term expansion of T cell clones and leads to tolerance, whereas targeting antigen to DCs in combination with DC maturation agents results to proliferation of T cell clones and therefore antigen-specific immunity (17, 30–32). In the present study, immunization of mice with rOVA-FLIPr without addition of exogenous adjuvant elicits potent CD4^+^ and CD8^+^ T cell responses (Figure 2) as well as antitumor ability (Figure 4). CD8^+^ DCs, not CD8^−^ DCs and pDCs, have a tendency toward maturation (Figure 7B) and ability of T cell activation (Figure 8) after injection of rOVA-FLIPr. These results indicate that rOVA-FLIPr can be targeted to CD8^+^ DCs and CD8^+^ DCs may play a critical role to mediate T cell activation.

Surface expression of CD127 has been identified as a useful marker for long-living memory T cells. Most importantly, it allows to distinguish between memory and effector T cells early after *in vivo* priming (20, 21). In addition, CD127 coordinates expression of CD27 and CD43 activation markers associated with capacity of memory T cells to mediate recall responses (22). Our results show that mice immunized with rOVA-FLIPr increase CD127^high^ CD27^high^ CD43^low^ and CD127^high^ CD27^high^ CD43^high^ T cell subsets (Figures 3A,B). The recall activity of antigen-specific memory T cell is further confirmed at week 17–19 after the first immunization (Figure 3C). These results suggest that immunization of mice with rOVA-FLIPr can induce long-living memory T cells with capacities to mount recall responses.

Protein/subunit vaccines typically require an exogenous adjuvant formulation to induce robust immune responses. However, the choice of adjuvant is very limited. Our results show that rVOA-FLIPr still maintains the FcγR binding capability. Importantly, immunization of mice with rOVA-FLIPr can induce robust T cell responses, CD8^+^ T cell-mediated antitumor immunity, and memory T cell responses with recall activity in the absence of exogenous adjuvant formulation. These findings support that FLIPr is a potent antigen delivery vector to augment antigen-specific responses. In addition, it has been shown that FLIPr can bind to different human FcγR isoforms and block IgG binding (18). These results suggest that this strategy can be applied to human vaccine development.

## Data Availability

All datasets generated for this study are included in the manuscript/Supplementary Files.

## Author Contributions

S-JL, C-HL, and H-WC contributed to the conception and design of the experiments. C-YC, C-CW, and Y-JC performed the experiments in the manuscript. S-JL, C-HL, and H-WC led the manuscript writing. All authors contributed to the analysis and interpretation of the results and participated to manuscript writing, editing, and critical reviewing.

### Conflict of Interest Statement

S-JL, C-HL, and H-WC are named on patent applications related to a method for enhancement of immune responses using an antigen fusion protein containing an antigen and an antagonist of an Fc gamma receptor. The remaining authors declare that the research was conducted in the absence of any commercial or financial relationships that could be construed as a potential conflict of interest.

## References

[1] Banchereau J Steinman RM. Dendritic cells and the control of immunity. Nature. (1998) 392:245–52. 10.1038/32588 9521319

[2] Banchereau J Briere F Caux C Davoust J Lebecque S Liu YJ. Immunobiology of dendritic cells. Annu Rev Immunol. (2000) 18:767–811. 10.1146/annurev.immunol.18.1.767 10837075

[3] Mellman I Steinman RM. Dendritic cells: specialized and regulated antigen processing machines. Cell. (2001) 106:255–8. 10.1016/S0092-8674(01)00449-4 11509172

[4] Apostolopoulos V Thalhammer T Tzakos AG Stojanovska L. Targeting antigens to dendritic cell receptors for vaccine development. J Drug Deliv. (2013) 2013:869718. 10.1155/2013/869718 24228179 PMC3817681

[5] Macri C Dumont C Johnston AP Mintern JD. Targeting dendritic cells: a promising strategy to improve vaccine effectiveness. Clin Transl Immunol. (2016) 5:e66. 10.1038/cti.2016.6 27217957 PMC4815026

[6] Amigorena S Bonnerot C. Fc receptor signaling and trafficking: a connection for antigen processing. Immunol Rev. (1999) 172:279–84. 10.1111/j.1600-065X.1999.tb01372.x 10631953

[7] Guilliams M Bruhns P Saeys Y Hammad H Lambrecht BN. The function of Fcgamma receptors in dendritic cells and macrophages. Nat Rev Immunol. (2014) 14:94–108. 10.1038/nri3582 24445665

[8] Heijnen IA van Vugt MJ Fanger NA Graziano RF de Wit TP Hofhuis FM. Antigen targeting to myeloid-specific human Fc gamma RI/CD64 triggers enhanced antibody responses in transgenic mice. J Clin Invest. (1996) 97:331–8. 10.1172/JCI118420 8567952 PMC507022

[9] Regnault A Lankar D Lacabanne V Rodriguez A Thery C Rescigno M. Fcgamma receptor-mediated induction of dendritic cell maturation and major histocompatibility complex class I-restricted antigen presentation after immune complex internalization. J Exp Med. (1999) 189:371–80. 10.1084/jem.189.2.371 9892619 PMC2192989

[10] den Haan JM Bevan MJ. Constitutive versus activation-dependent cross-presentation of immune complexes by CD8(+) and CD8(-) dendritic cells *in vivo*. J Exp Med. (2002) 196:817–27. 10.1084/jem.20020295 12235214 PMC2194052

[11] Akiyama K Ebihara S Yada A Matsumura K Aiba S Nukiwa T. Targeting apoptotic tumor cells to Fc gamma R provides efficient and versatile vaccination against tumors by dendritic cells. J Immunol. (2003) 170:1641–8. 10.4049/jimmunol.170.4.1641 12574326

[12] Tobar JA Gonzalez PA Kalergis AM. Salmonella escape from antigen presentation can be overcome by targeting bacteria to Fc gamma receptors on dendritic cells. J Immunol. (2004) 173:4058–65. 10.4049/jimmunol.173.6.4058 15356155

[13] Flinsenberg TW Compeer EB Koning D Klein M Amelung FJ van Baarle D. Fcgamma receptor antigen targeting potentiates cross-presentation by human blood and lymphoid tissue BDCA-3+ dendritic cells. Blood. (2012) 120:5163–72. 10.1182/blood-2012-06-434498 23093620

[14] Rafiq K Bergtold A Clynes R. Immune complex-mediated antigen presentation induces tumor immunity. J Clin Invest. (2002) 110:71–9. 10.1172/JCI0215640 12093890 PMC151032

[15] Schuurhuis DH Ioan-Facsinay A Nagelkerken B van Schip JJ Sedlik C Melief CJ. Antigen-antibody immune complexes empower dendritic cells to efficiently prime specific CD8+ CTL responses *in vivo*. J Immunol. (2002) 168:2240–6. 10.4049/jimmunol.168.5.2240 11859111

[16] van Montfoort N Mangsbo SM Camps MG van Maren WW Verhaart IE Waisman A. Circulating specific antibodies enhance systemic cross-priming by delivery of complexed antigen to dendritic cells *in vivo*. Eur J Immunol. (2012) 42:598–606. 10.1002/eji.201141613 22488363

[17] Lehmann CHK Baranska A Heidkamp GF Heger L Neubert K Luhr JJ. DC subset-specific induction of T cell responses upon antigen uptake via Fcgamma receptors *in vivo*. J Exp Med. (2017) 214:1509–28. 10.1084/jem.20160951 28389502 PMC5413326

[18] Stemerding AM Kohl J Pandey MK Kuipers A Leusen JH Boross P. *Staphylococcus aureus* formyl peptide receptor-like 1 inhibitor (FLIPr) and its homologue FLIPr-like are potent FcgammaR antagonists that inhibit IgG-mediated effector functions. J Immunol. (2013) 191:353–62. 10.4049/jimmunol.1203243 23740955

[19] Wu CC Liu SJ Chen HW Shen KY Leng CH. A Toll-like receptor 2 agonist-fused antigen enhanced antitumor immunity by increasing antigen presentation and the CD8 memory T cells population. Oncotarget. (2016) 7:30804–19. 10.18632/oncotarget.9001 27127171 PMC5058719

[20] Kaech SM Tan JT Wherry EJ Konieczny BT Surh CD Ahmed R. Selective expression of the interleukin 7 receptor identifies effector CD8 T cells that give rise to long-lived memory cells. Nat Immunol. (2003) 4:1191–8. 10.1038/ni1009 14625547

[21] Huster KM Busch V Schiemann M Linkemann K Kerksiek KM Wagner H. Selective expression of IL-7 receptor on memory T cells identifies early CD40L-dependent generation of distinct CD8+ memory T cell subsets. Proc Natl Acad Sci USA. (2004) 101:5610–5. 10.1073/pnas.0308054101 15044705 PMC397444

[22] Hikono H Kohlmeier JE Takamura S Wittmer ST Roberts AD Woodland DL. Activation phenotype, rather than central- or effector-memory phenotype, predicts the recall efficacy of memory CD8+ T cells. J Exp Med. (2007) 204:1625–36. 10.1084/jem.20070322 17606632 PMC2118640

[23] Hashimoto D Miller J Merad M. Dendritic cell and macrophage heterogeneity *in vivo*. Immunity. (2011) 35:323–35. 10.1016/j.immuni.2011.09.007 21943488 PMC4520532

[24] Schraml BU Reis e Sousa C. Defining dendritic cells. Curr Opin Immunol. (2015) 32:13–20. 10.1016/j.coi.2014.11.001 25553392

[25] Tacken PJ Figdor CG. Targeted antigen delivery and activation of dendritic cells *in vivo*: steps towards cost effective vaccines. Semin Immunol. (2011) 23:12–20. 10.1016/j.smim.2011.01.001 21269839

[26] Segura E Villadangos JA. Antigen presentation by dendritic cells *in vivo*. Curr Opin Immunol. (2009) 21:105–10. 10.1016/j.coi.2009.03.011 19342210

[27] den Haan JM Lehar SM Bevan MJ. CD8(+) but not CD8(-) dendritic cells cross-prime cytotoxic T cells *in vivo*. J Exp Med. (2000) 192:1685–96. 10.1084/jem.192.12.1685 11120766 PMC2213493

[28] Pooley JL Heath WR Shortman K. Cutting edge: intravenous soluble antigen is presented to CD4 T cells by CD8- dendritic cells, but cross-presented to CD8 T cells by CD8+ dendritic cells. J Immunol. (2001) 166:5327–30. 10.4049/jimmunol.166.9.5327 11313367

[29] Smith CM Belz GT Wilson NS Villadangos JA Shortman K Carbone FR. Cutting edge: conventional CD8 alpha+ dendritic cells are preferentially involved in CTL priming after footpad infection with herpes simplex virus-1. J Immunol. (2003) 170:4437–40. 10.4049/jimmunol.170.9.4437 12707318

[30] Dudziak D Kamphorst AO Heidkamp GF Buchholz VR Trumpfheller C Yamazaki S. Differential antigen processing by dendritic cell subsets *in vivo*. Science. (2007) 315:107–11. 10.1126/science.1136080 17204652

[31] Bonifaz L Bonnyay D Mahnke K Rivera M Nussenzweig MC Steinman RM. Efficient targeting of protein antigen to the dendritic cell receptor DEC-205 in the steady state leads to antigen presentation on major histocompatibility complex class I products and peripheral CD8+ T cell tolerance. J Exp Med. (2002) 196:1627–38. 10.1084/jem.20021598 12486105 PMC2196060

[32] Hawiger D Inaba K Dorsett Y Guo M Mahnke K Rivera M. Dendritic cells induce peripheral T cell unresponsiveness under steady state conditions *in vivo*. J Exp Med. (2001) 194:769–79. 10.1084/jem.194.6.769 11560993 PMC2195961

