# Supplementary material for: Delivery of Antigen to CD8+ Dendritic Cells by Fusing Antigen With Formyl Peptide Receptor-Like 1 Inhibitor Protein Induces Antitumor Immunity
Source: Front Immunol. 2019 Aug 2;10:1839. doi: 10.3389/fimmu.2019.01839 (PMC6688046; doi:10.3389/fimmu.2019.01839)
Supplement: Supplementary file 1 [file Data_Sheet_1.pdf]

Supplementary Material

Delivery of antigen to CD8+ dendritic cells by fusing antigen with formyl peptide receptor-like 1 inhibitor protein induces antitumor immunity

Chen-Yi Chiang, Chiao-Chieh Wu, Yi-Jyun Chen, Shih-Jen Liu, Chih-Hsiang Leng, Hsin-Wei Chen*

*** Correspondence:** Hsin-Wei Chen (chenhw@nhri.org.tw)

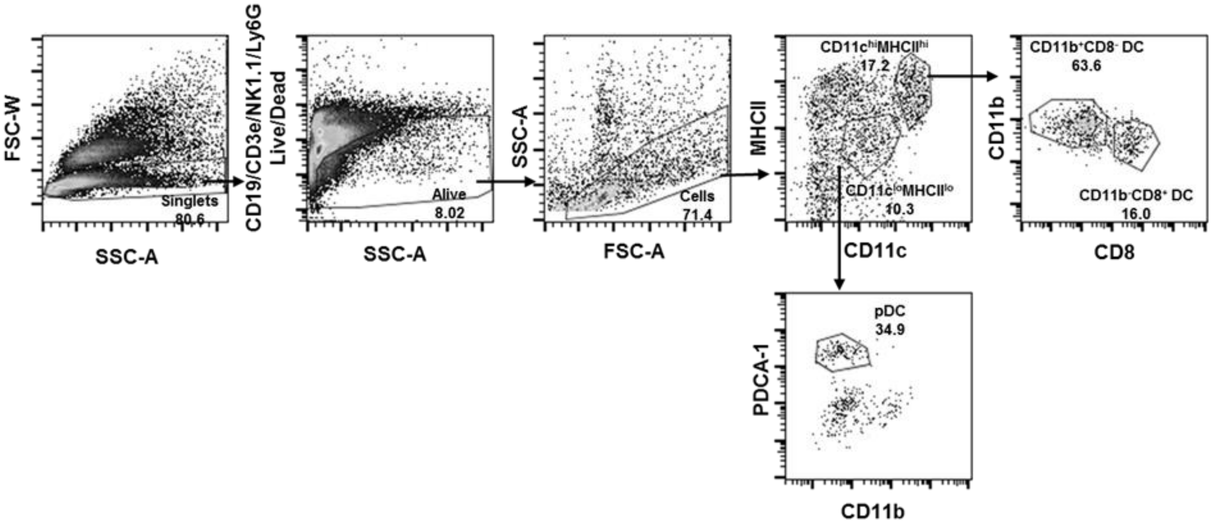

Supplementary Figure 1. Gating strategy of DC subpopulations. Spleen or lymph node single-cell suspensions were prepared from C57BL/6 mice. Cells were stained with CD3, CD8, CD11b, CD11c, CD19, Ly6G, MHC II, NK1.1, PDCA-1, and Live/Dead fixable dead cell stains. Singlets were gated by FSC-W/SSC-A followed by exclusion of T cells, B cells, NK cells, and neutrophils. CD11c^{+} cells were further analyzed for CD11c^{high}MHC II^{high}CD11b^{-}CD8^{+} DCs (CD8^{+} DCs), CD11c^{high}MHC II^{high}CD11b^{+}CD8^{-} DCs (CD8^{-} DCs), and CD11c^{low}MHC II^{low}CD11b^{-}PDCA-1^{+} DCs (pDCs).

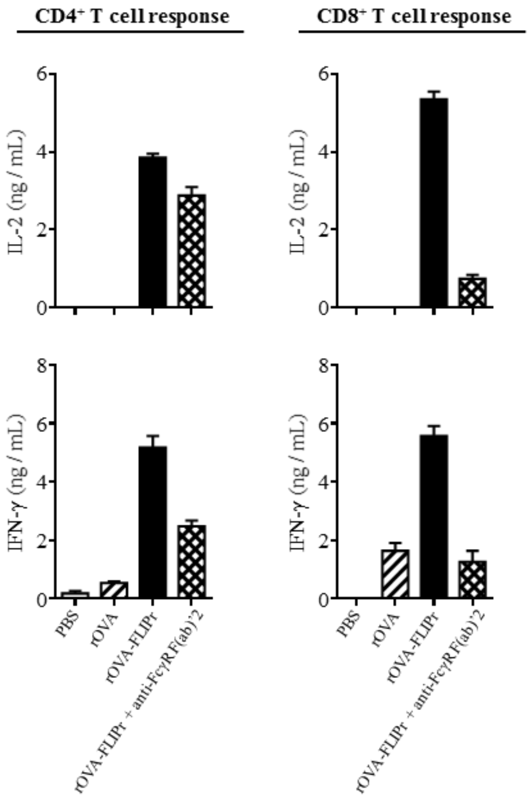

Supplementary Figure 2. Targeting of rOVA-FLIPr to dendritic cells is via FcγR-dependent pathways. The $CD11c^+$ cells were enriched from splenocytes of C57BL/6 mice. $CD11c^+$ cells (1×10^5 /ml) incubated with or without rabbit F(ab)'$_2$ antibodies against FcγR1, 2b, 3 or 4 (10 μg/ml each F(ab)'$_2$ antibody) at 37°C for 30 min. Sequentially, $CD11c^+$ cells were pulsed with rOVA or rOVA-FLIP (10 μg/ml) at 37°C for 1.5 hrs. PBS treated-$CD11c^+$ cells were used as a negative control. The cultured cells were washed with culture medium, and co-cultured with OT-1 or OT-2 T cells for 24 or 40 hrs, respectively. Levels of IL-2 and IFN-γ in the supernatants were determined by ELISA. Data represent mean ± SEM from triplicate wells. The results are one of two representative experiments.
